# Osteomyelitis Caused by *Candida glabrata* in the Distal Phalanx

**DOI:** 10.1155/2014/962575

**Published:** 2014-08-24

**Authors:** Shunichi Toki, Naohito Hibino, Koichi Sairyo, Mitsuhiko Takahashi, Shinji Yoshioka, Masahiro Yamano, Tatsuhiko Henmi

**Affiliations:** ^1^Tokushima Prefecture Naruto Hospital, 32 Kotani, Kurosaki, Muya-cho, Naruto-shi, Tokushima 772-8503, Japan; ^2^National Hospital Organization Kochi National Hospital, 1-2-25 Asakuranishi-machi, Kochi 780-8081, Japan; ^3^Institute of Health Biosciences, The University of Tokushima Graduate School Institution, Kuramoto-cho 3-18-15, Tokushima-shi, Tokushima 770-8503, Japan

## Abstract

Osteomyelitis caused by *Candida glabrata* is rare and its optimal treatment is unknown. Here we report a case of osteomyelitis caused by *C. glabrata* in the distal phalanx in a 54-year-old woman. Despite partial resection of the nail and administering a 1-month course of antibiotics for paronychia, the local swelling remained and an osteolytic lesion was found. *C. glabrata* osteomyelitis of the distal phalanx was later diagnosed after curettage. Thereafter, the patient was treated with antifungal agents for 3 months. The infection eventually resolved, and radiological healing of the osteolytic lesion was achieved. Antifungal susceptibility testing should be performed in the case of osteomyelitis caused by nonalbicans Candida species, due to their resistance to fluconazole.

## 1. Introduction

Invasive candidiasis has increased in prevalence with the increase in the elderly population, but* Candida* osteomyelitis is relatively rare [1]. The most frequent mechanism of bone infection in* Candida* osteomyelitis is hematogenous dissemination, and the most commonly affected sites in adults are the vertebra, ribs, and sternum [2, 3]. We report a case of osteomyelitis caused by* C. glabrata* affecting the distal phalanx of the middle finger, which was successfully treated with operative debridement followed by a 3-month course of antifungal treatment.

## 2. Case Report

A 54-year-old woman with a treatment history of hypertension presented with progressive pain in the left middle finger, which had persisted for the past 4 months. She worked as an esthetician scrubbing scurf and had been treated by a local doctor when her pain first developed. She underwent partial resection for an ingrown nail and received antibiotics treatment for paronychia of the finger. When her condition did not improve, she was referred to our department because of residual pain and osteomyelitis suspected from local inflammatory findings.

**Figure 1:**
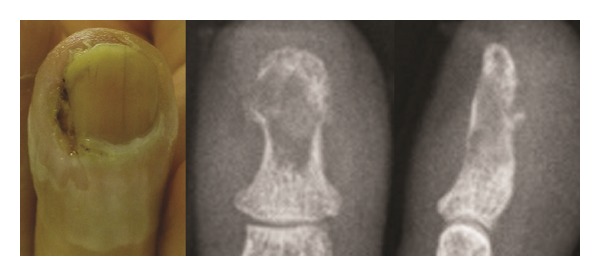
Anteroposterior and lateral radiographs at the first presentation.

**Figure 2:**
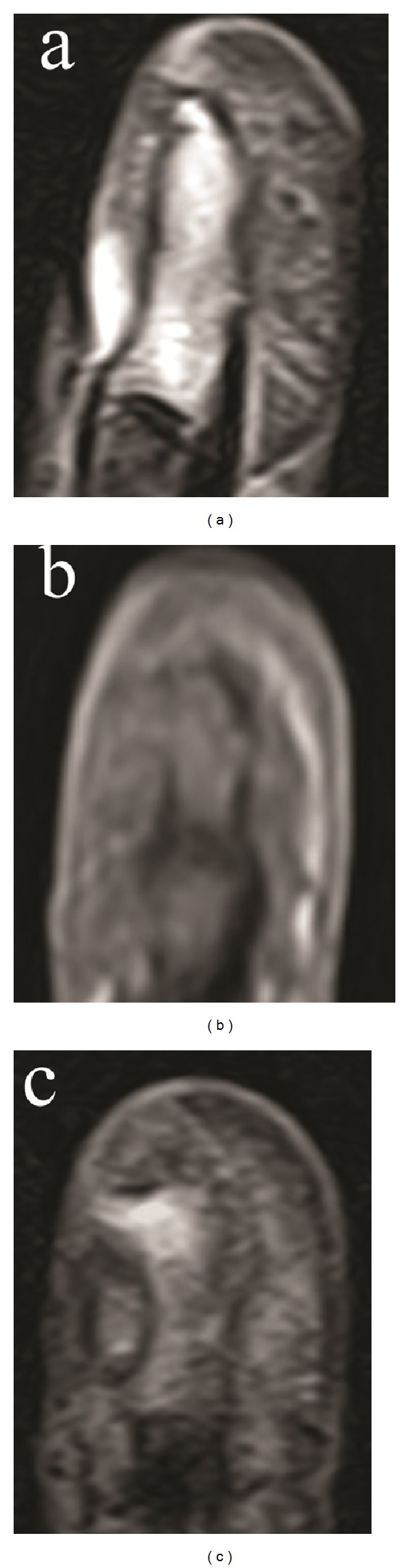
Sagittal view of short time inversion recovery magnetic resonance images (MRI) (a) and coronal views of T1 weighted (b) and T2 weighted (c) MRI.

The fingertip was swollen with scales around the nail wall (Figure 1). Radiography revealed osteolysis and disruption of the ulnar cortical bone of the distal phalanx. Her white blood cell count was 5.3 × 10^3^/mm^3^ and C-reactive protein was 0.03 mg/dL. Magnetic resonance imaging of the finger revealed diffuse high signal intensity in the bone marrow of the distal phalanx and a subcutaneous fluid-like lesion with an isosignal intensity area on T1 weighted images and a high signal intensity area on T2 weighted images (Figure 2).

**Figure 3:**
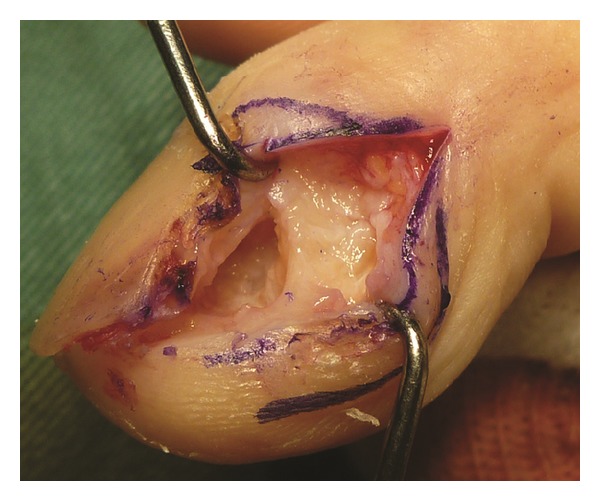
Intraoperative findings showing a partial defect of the ulnar cortical bone of the distal phalanx.

Debridement and curettage were performed via the ulnar midlateral approach. A partial defect of the cortical bone, through which surrounding subcutaneous tissue is connected to the bone marrow, was noted. A small amount of pus-like fluid was aspirated from a cavity in the distal phalanx (Figure 3). The distal interphalangeal joint was immobilized postoperatively with a splint for 2 months.

**Figure 4:**
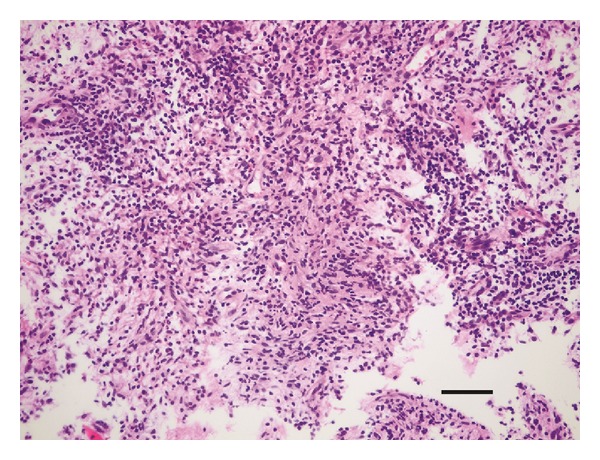
Histological findings of the lesion (hematoxylin eosin staining). The scale bar indicates 50 *μ*m.

**Figure 5:**
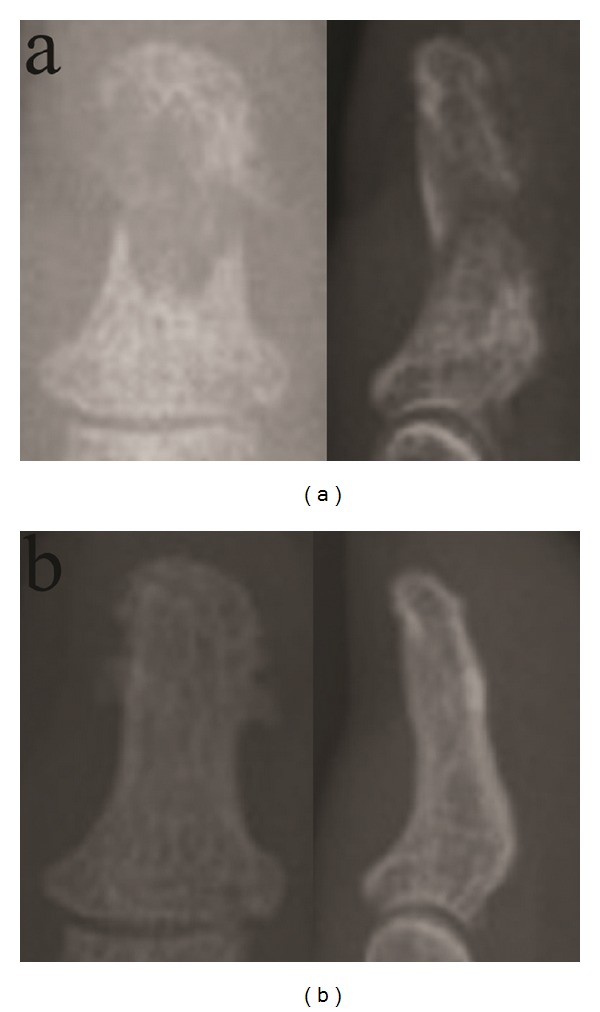
Anteroposterior and lateral radiographs at 1 month (a) and 10 months (b) after surgery.

Flomoxef sodium (2,000 mg/day) was given intravenously for 7 days postoperatively.* C. glabrata* was detected in fluid culture, and histological findings showed a granuloma with lymphocyte infiltration and a small quantity of spores (Figure 4). Intravenous itraconazole (400 mg/day) was given for 3 days, followed by intravenous fluconazole (200 mg/day) for 5 days due to the side effects of itraconazole. The patient was further treated with oral flucytosine (3,000 mg/day) for 3 months. Her clinical findings improved and her pain disappeared within 1 month after surgery, and she returned to work 2 months after surgery. At the final followup (10 months after surgery), new bone formation was seen at the bone defect in the distal phalanx and the cortical bone disruption was healed with no recurrence of inflammation (Figure 5).

## 3. Discussion

*Candida* osteomyelitis is relatively rare, with most reports limited to case descriptions and a few case series. On the other hand invasive candidiasis is becoming more prevalent as medicine continues to change, including the extensive use of prophylactic antifungal agents, broad-spectrum antibacterial agents, and medical devices (e.g., chronic indwelling intravascular catheters) and the patient population continues to change [1]. In the case of* Candida* osteomyelitis, the mechanisms of bone infection are classified as follows: hematogenous dissemination (67%), direct inoculation (25%), and contiguous infection (9%) [2]. The most common infecting species are* C. albicans* (69%),* C. tropicalis* (15%), and* C. glabrata* (8%) [3], and the most commonly affected sites in adults are the vertebra, ribs, and sternum.* Candida* osteomyelitis of the phalanx is relatively rare [2, 4, 5].

*Candida* is now recognized to be a cause of deep-site infections, including osteomyelitis, in patients with risk factors such as diabetes mellitus, deficient cell-mediated immunity, neutropenia, steroid use, central catheter insertion, total parenteral nutrition, and a prior history of broad-spectrum antimicrobials use.* C. glabrata* is an increasingly important cause of candidemia, now accounting for about one quarter of* Candida* bloodstream infections in the United States [6]. However, osteomyelitis caused by* C. glabrata* is a relatively rare condition and, to our knowledge, only a few cases have been reported [7–10]. The present case of osteomyelitis was caused by* C. glabrata* affecting the distal phalanx of the middle finger but without any of those risk factors. In addition, chronic paronychia caused by Candida species occurs generally among workers with the exposure to excessive moisture [11]. Thus we guess that* C. glabrata* initially caused paronychia which further developed into osteomyelitis of the phalanx through contiguous infection mechanism over 4 months before the consultation.

Although susceptibility testing to antifungal agents was not performed in this case, osteomyelitis was successfully cured with surgical curettage followed by antifungal treatment (flucytosine) for 3 months. Flucytosine has comparatively low toxicity and was given without any combination dosages of other antifungal agents to avoid side effects. Compared with patients infected with* C. albicans*, those with nonalbicans Candida species including* C. glabrata* are more likely to have received an inadequate dose of fluconazole as initial therapy because* C. glabrata* often exhibits resistance to fluconazole [12]. Therefore we emphasize to carry out susceptibility testing to obtain accurate MIC values and to choose the antifungal agents, in which amphotericin B would be the first choice, in the case of osteomyelitis caused by nonalbicans Candida species [3, 7–10].

Another study reported a severe case of osteomyelitis for 6 months caused by* C. guilliermondii* in a 57-year-old patient that resulted in partial amputation of the affected finger [4], and the strains isolated in culture were highly resistant to fluconazole and itraconazole. As a result, the symptoms progressed, and a deep and painful ulcer with radiologic signs of progressing acroosteolysis required partial amputation at the distal interphalangeal joint. Because bone and joint fungal infection are difficult to diagnose and as delayed diagnosis can lead to a progression of infection, fungal infection should be considered as a differential diagnosis in cases presenting with prolonged or chronic refractory inflammation, even in the digits.
